# Reproductive Toxicity of Zishen Yutai Pill in Rats: The Fertility and Early Embryonic Development Study (Segment I)

**DOI:** 10.1155/2016/3175902

**Published:** 2016-12-12

**Authors:** Li Zhou, Qiuling Huang, Rong Wang, Jie Zhou, Aicui Ma, Liming Chong, Yubing Wu, Yong Wang, Li Xu, Ying Chen, Yuling Jia, Bo Gui, Zuyue Sun

**Affiliations:** ^1^National Evaluation Centre for the Toxicology of Fertility Regulating Drug, Department of Pharmacology and Toxicology, Shanghai Institute of Planned Parenthood Research, Shanghai 200032, China; ^2^Guangzhou Baiyunshan Zhongyi Pharmaceutical Co., Ltd., Guangzhou, China

## Abstract

*Purpose*. This study was aimed to investigate the reproductive toxicity of Zishen Yutai Pill (ZYP) on fertility and early embryonic development in rats.* Methods*. SD rats were randomly divided into 5 groups: vehicle control group (distilled water, i.g.), positive control group (80 mg/kg of cyclophosphamide, i.p.), and three ZYP-treated groups (3, 6, and 12 g/kg/d, i.e., 12x, 24x, and 48x clinical doses, i.g.). The high dose was set as the maximum gavage dosage.* Results*. Cyclophosphamide showed diverse hazards, such as decreased weight of male reproductive organs and sperm density (*P* < 0.05). However, there were no obvious effects of ZYP on physical signs, animal behavior, and survival rate, as well as on weight and food intake during the premating and gestation periods. Importantly, there were no significant adverse effects of ZYP on indexes of copulation, fecundity and fertility indexes, weights and coefficients of male reproductive organs, epididymal sperm number and motility, estrous cycle, preimplantation loss rate, and implantation rate. Besides, the numbers of live and resorbed fetuses per litter were not significantly altered.* Conclusions*. ZYP had no reproductive toxicities on fertility and early embryonic development in rats at 48x equivalent clinical doses.

## 1. Introduction

Zishen Yutai Pill (ZYP) is a Chinese herbal medicine compound preparation that contains fifteen herb formulas [1, 2]. It is recently reported to enhance endometrial receptivity and prevent recurrent miscarriage [1] and strengthen the function of spleen and kidney [3]. Also, it is an effective medicine [4] to treat luteal phase defect menstrual disorder [5], asthenospermia [6], and spleen-kidney deficiency type patients of sterility [7].

Several researches have been carried out to assess the possible hazards of ZYP during treatment in regard to menstruation, endocrine indices, body temperature, endometrium, abortion rate, abdominal pain, and vaginal bleeding [8, 9]. However, considering that this drug is often prescribed to both women and men of childbearing potential [10], it is still far from enough to expatiate its adverse effects and safety. Herein, we begin to address this issue for ZYP in three reproductive toxicology studies in succession, including its effects on fertility and early embryonic development in rats (segment I), embryo-fetal development in rats and rabbits (segment II), and prenatal and postnatal development in rats (segment III). In the embryo-fetal development study, we found that only mild maternal toxicities of ZYP were detected in rats at the dose of 24 g/kg/d and were limited to decreased extra-uterine weight gain and food intake, and there was no reproductive toxicology in rabbits even at the maximum administration dose [11]. The present preclinical study further reports the effects of ZYP on fertility and early embryonic development in male and female rats, which provides needed preclinical safety evidence to support its clinical usage.

## 2. Methods

### 2.1. Test Article

The dosage form of ZYP is water-honeyed pill. To produce the water-honeyed pill, the 15 herbal materials (Fructus Amomi, 72 g; Dipsaci Radix, 480 g;* Cuscuta sinensis *Lam., 720 g; Radix Rehmanniae Preparata, 480 g; Artemisiae Argyi Folium, 144 g; Herba Taxilli, 480 g; Genseng, 48 g;* Atractylodes macrocephala*, 240 g; Colla Corii Asini, 33 g;* Cornu cervi degelatinatum*, 144 g; Polygonum Multiflorum, 480 g;* Eucommia ulmoides*, 288 g;* Codonopsis *Radix, 576 g; Fructus Lycii, 192 g; and* Morinda officinalis*, 192 g) are crushed to powder, mixed, and then molded into pills together with honey and water [7].

In the present study, the test drug was the raw power of ZYP provided by Guangzhou Baiyunshan Zhongyi Pharmaceutical Co. Ltd. (Lot Number: 20120401, Guangzhou, Guangdong, China). Dosing solutions were prepared by dissolving in distilled water which was given as vehicle control. Cyclophosphamide (Lot Number: 20110111, Sunray pharmaceutical Co., Ltd., Suzhou, Jiangsu, China) was used as positive control of this reproductive toxicity study and was prepared with saline solution (Lot Number: 110827k5, Fengyuan Pharmaceutical Co., Ltd., Hefei, Anhui, China). All solutions were prepared daily.

### 2.2. Animals

The specific-pathogen-free (SPF) Sprague-Dawley rats purchased from SIPPR-B&K Laboratory Animal Co. Ltd. (Shanghai, China) were used. Animal dosing and toxicology analyses were performed at the National Evaluation Centre for the Toxicology of Fertility Regulating Drug (Department of Pharmacology and Toxicology, Shanghai Institute of Planned Parenthood Research, Shanghai, China) and conducted according to the drug reproductive toxicity study technical guidelines recommended by China Food and Drug Administration (CFDA) [12]. This study was approved by the Institutional Animal Care and Use Committee (IACUC-20130315-01).

### 2.3. Dosage Set of ZYP

The tested doses of ZYP in the present study were calculated based on the FAD-recommended formula for traditional Chinese medicine dose translation between human and experimental animal: *d*_*B*_ = *d*_*A*_ × *R*_*B*_/*R*_*A*_ × (*W*_*A*_/*W*_*B*_)^1/3^ [13]. In this formula, *d*_*A*_ stands for the human dose, while *d*_*B*_ stands for the animal dose; *R* means the coefficient of body weight (100 for human and 90 for rat); and *W* is the standard body weight (60 kg for human and 200 g for rat). As the clinical dose of ZYP for human is 0.25 g/kg/d, 1x clinical dose for rat is 1.5 g/kg/d.

Our preliminary experiment indicated that the maximum concentration of ZYP was 0.40 g/mL and the maximum administration volume was 30 mL/kg/time. Based on the recommended guidelines by CFDA [12], the high dose was set as the maximum gavage dosage (12 g/kg/d, i.e., 48x clinical dose) for rats, and thus 3 and 6 g/kg/d of ZYP (i.e., 12x and 24x clinical doses) were tested as low and moderate doses to investigate its effect on the fertility and early embryonic development in rats.

### 2.4. Experimental Designs

A total of 125 male and 125 female rats were used in this study. Male and female rats were separately housed in cages in an SPF animal room which was maintained at 20–26°C and 40%–70% relative humidity with a 12/12 h light and dark cycle. All rats were maintained with free access to sterile water and food.

Males (aged 50–60 d, weighting 117.0–153.2 g at the beginning of administration) were obtained 7 weeks earlier than females (aged 90–100 d, weighting 188.4–233.2 g at the beginning of administration). Following an acclimation period of 5 days, rats were assigned to one of the following five groups (25 animals/sex/group) based on a random block design taking weight as block criterion: vehicle control group, positive control group, and three different doses of ZYP-treated groups.

The day of first administration was defined as D_1_. Prior to cohabitation, male and female rats in the vehicle control and ZYP-treated groups were intragastrically dosed for 9 and 2 consecutive weeks at a dose volume of 30 mL/kg, respectively. Then, each male was housed individually with a female within the same group for mating. Vaginal smear examination was conducted for the female rats at 8:00–9:30 am during the period of mating until sperm or vaginal plug was detected. The day was defined as Gestation Day 0 (GD_0_). Treatment lasted throughout the mating period for males and continued until GD_7_ for females. Rats in the positive control groups were intraperitoneally injected with cyclophosphamide at 80 mg/kg on D_1_, as a bolus injection. Pregnant rats were subjected to a caesarean section on GD_14_, and males were sacrificed on GD_0_.

### 2.5. Outcome Measures

Physical sign, behavior, and survival of rats were closely observed and recorded. Rats were weighted twice a week before mating and the females were also weighed on GD_0_, GD_3_, GD_7_, GD_10_, and GD_14_. Meanwhile, food intakes were measured during the first days before mating for all rats and during GD_0-1_, GD_6-7_, and GD_13-14_ for the pregnant rats.

Indexes of copulation, fecundity, and fertility were calculated based on the number of cohabited, copulated, and pregnant females. Furthermore, terminal inspections were taken for male and female rats, respectively. After they were sacrificed, testis, epididymis, foreskin gland, prostate, seminal vesicles, anal sphincter, and levator ani muscle were separated and weighted. Additionally, motility, count, and morphology of sperm were analyzed by Computer Assisted Sperm Analysis (Hamilton Thorne TOX IVOS). Besides, motile sperm density, progressive concentration, motility (%), rapid (%), average path velocity (VAP, m/s), straight-line (rectilinear) velocity (VSL, m/s), amplitude of lateral head displacement (ALH, *μ*m), curvilinear velocity (VCL, m/s), beat-cross frequency (BCF, HZ), linearity (LIN, %), and elongation (ELO, %) were examined for assessing the motility of sperm by using Computer Assisted Sperm Analysis (Hamilton Thorne TOX IVOS) and VERSION 12 Toxicology Software. For female rats, corpora lutea count, total weight of the gravid uterus, and numbers of live and resorbed fetuses were recorded. Implantation number, preimplantation mortality, postimplantation mortality, and average implantation rate were also calculated and recorded.

All outcomes were measured by a researcher who was blinded to the treatment group.

### 2.6. Statistical Analysis

Quantitative data were presented as mean ± standard deviation (SD) and the differences were analyzed by univariate variance analysis followed by Dunnett test. Qualitative data were expressed as percentages and analyzed using *χ* ^2^ test. All the analyses were conducted by using SPSS version 19.0 (SPSS Inc., Chicago, IL, USA). *P* value less than 0.05 was considered as statistically different.

## 3. Results

### 3.1. Changes in Physical Signs, Behavior, and Survival

No animals died due to treatment before being sacrificed. Rats in the ZYP-treated groups showed normal behaviors and physical signs such as dense and shiny hairs and spiritual eyes. No rapid respiration, prone positioning, and lethargy were observed. In the positive control group, however, bloody secretions were observed around eyes and nasal of several rats on the 5th or 6th day following cyclophosphamide administration. The secretions existed for 3 days in females and 6 days in males. Additionally, hair loss was observed in most males on the 6th day following treatment and lasted for 2–7 days.

### 3.2. Changes in Weight and Food Intake of Male Rats

**Figure 1:**
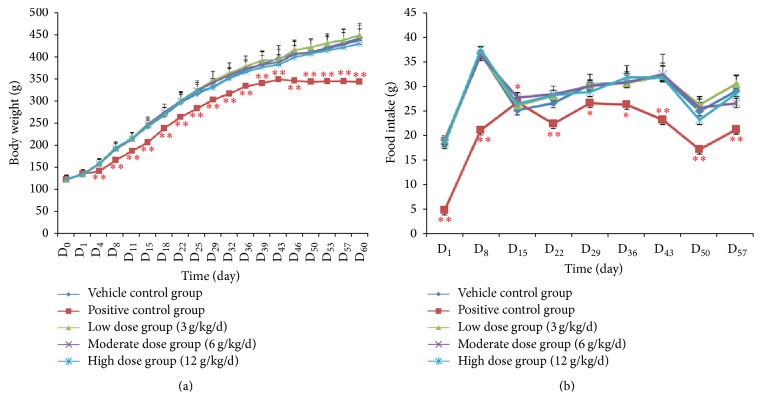
Changes in body weight (a) and food intake (b) for male rats during the premating period. ^*∗*^ *P* < 0.05 and ^*∗∗*^ *P* < 0.01 compared with the vehicle control group.

Weight and food intake of male rats were determined during the premating period. No significant differences in weight existed between various doses of ZYP-treated groups and vehicle control group during the experiment (Figure 1(a)). Also, no significant decrements in food consumption attributed to ZYP administration were observed (*P* > 0.05, Figure 1(b)), except that food intake was increased on D_15_ in moderate dose group (27.6 ± 0.7 versus 25.1 ± 0.5, *P* < 0.05). For rats in the positive control group, the weight was significantly decreased compared with that in the vehicle control group from D_4_ to D_60_ (*P* < 0.01, Figure 1(a)). Along with the decreased body weight, the food intake of rats also significantly declined after cyclophosphamide treatment (*P* < 0.05, Figure 1(b)).

### 3.3. Reproductive Toxicity Analysis for Male Rats

Overall, the copulation index, fecundity index, and fertility index were similar and without any statistical differences among the vehicle control group, positive control group, and three doses of ZYP-treated groups (Table 1, *P* > 0.05). In addition, mating period was not extended (Table 1, *P* > 0.05).

No significant differences existed in weights and coefficients of testis, epididymis, preputial glands, prostate, seminal vesicles, anal sphincter, and levator ani muscle between the vehicle control group and ZYP-treated groups (Table 2, *P* > 0.05). However, the weights of body, prostate, seminal vesicles, anal sphincter, and levator ani muscle were tremendously decreased, and coefficients of testis, epididymis, and sphincter were notably increased in the positive control group, as compared with the vehicle control group (Table 2, *P* < 0.05).

The changes in epididymal sperm number or motility are summarized in Table 3. ZYP treatment did not markedly affect the sperm motility way (ALH, STR, and LIN), the vitality of sperm (VAP, VSL, VCL, and BCF), and occurrence of sperm deformity (*P* > 0.05). Compared with the vehicle control group, count and density of sperm were distinctly decreased, whereas the count of sperm deformity was significantly increased (69.7 ± 5.5 versus 14.1 ± 2.0, *P* < 0.05) in the positive control group.

### 3.4. Changes in Weight and Food Intake of Females

Weight, maternal weight, and the gain of extra-uterine weight were recorded without significant differences among these five groups (Table 4, *P* > 0.05). During the premating period, food intake was significantly increased on D_8_ (20.4 ± 1.7 versus 17.0 ± 1.8, *P* < 0.05) in the high dose group but sharply decreased on D_1_ (8.2 ± 1.8 versus 15.4 ± 1.4, *P* < 0.05) in the positive control group (Table 4). However, ZYP and cyclophosphamide had no significant effects on food intakes of pregnant rats (*P* > 0.05, Table 4).

### 3.5. Reproductive Toxicity Analysis in Female Rats

In addition to the indexes of copulation, fecundity, and fertility (Table 1), the unaltered estrous cycle, preimplantation loss rate, and implantation rate (Table 5) also demonstrated that ZYP had no obvious effects on the fertility of female rats. Besides, the pregnancy outcomes including live and resorbed fetuses per litter were not significantly influenced by ZYP or cyclophosphamide (Table 5, *P* > 0.05).

## 4. Discussion

ZYP has rich clinical experiences in treating multiple diseases such as infertility and miscarriage. Particularly, satisfactory efficacy of 90.0% [14] to 94.35% [2] was achieved for patients with habitual abortion and threatened abortion, and no adverse effect was observed in the newborns during a 60-day follow-up period [14]. Despite these findings, the safety and possible toxicology of ZYP are generally poorly investigated, as well as the therapeutic mechanism. Hence, we performed this segment I reproductive toxicity research for ZYP. To our limited knowledge, this is the first time to study the reproductive toxicity of ZYP on fertility and early embryonic development in rats. Here, cyclophosphamide was taken as a positive control because it could interfere with oogenesis and spermatogenesis, which caused infertility in both genders [15, 16]. According to the results, secretions were observed around eyes and nasal of several rats, and weight and food intake were significantly decreased after cyclophosphamide treatment. Consistent with the previous studies, cyclophosphamide had negative effects on the fertility of male rats by reducing the weights of prostate, seminal vesicles, levator ani muscle, and the anal sphincter as well as downregulating the concentration and count of sperm [17]. Lower corpora lutea, number of implantations, implantation rate, and live births rate were also found in the cyclophosphamide treated group. All these results certified the effectiveness and reliability of our experiments.

ZYP had no obvious effects on the physical signs, animal behavior, and survival rate, as well as body weight. Although the food consumption was slightly undulated following the administration of ZYP, no dose-depended tendency was found. The reduction of the absolute weight for the testes, prostate, seminal vesicle, and epididymis in rats indicated the reproductive toxic on these organs [18]. Testicular mass was considered as a valuable index for the reproductive toxicity in male animals and the testicular mass decreasing was consistent with the germ cells elimination [19]. According to our study, no significant differences were found in weights and coefficients of organs (testis, epididymis, foreskin gland, prostate, seminal vesicles, anal sphincter, and levator ani muscle) between the ZYP groups and vehicle control group. Semen volume, concentration, morphology, sperm motility, and viability were considered as the five most critical factors for the reproduction potential in males [20, 21]. In this reproductive toxicity evaluation test, we found no significant changes in sperm motility way (ALH, STR, and LIN) and vitality of sperm (VAP, VSL, VCL, and BCF), and no sperm deformity was found after ZYP treatment. These sperm parameters were considered to be prime markers for the epididymal maturation and testicular spermatogenesis, which were important indicators for the male fertility [18, 22]. Furthermore, previous study had shown that ZYP could improve uterine receptivity [1]. Genseng could improve the sperm quality and treat infertility, indicating the multifaceted effects on male reproductive function [23]. Dipsaci Radix had been proved to treat pregnant disorders and prevent miscarriage [24]. Radix Rehmanniae Preparata was used to treat gynaecological disorders [25]. Colla corii Asini and Fructus Lycii were reported to be served as drugs and food [26, 27]. Fructus Amomi was also commonly used as a food spice [27]. These data suggested that these herbal materials might be safe and ZYP might be beneficial in reproductive function. Therefore, ZYP could be safely used for the males without effects on their fertility at the clinical dose.

Estrous cycle, ovarian and uterine weight, intra-uterine fetal weight, and even the average number of corpora lutea were common factors for fertility evaluation of females [28, 29]. Corpora lutea was closely associated with fertility index, and fewer corpora lutea had been found to be associated with the reducing of fertility [30, 31]. In addition, decreasing ovarian and uterine weight had negative effects on the reproductive function in female rats [32]. As shown in our study, no significant differences of fertility evaluation factors were found in female rats after ZYP treatment, which suggested that ZYP treatment had no adverse pregnancy outcomes or reproductive toxicity on the fertility of females. Meanwhile, ZYP treatment had no effects on the early embryonic development as no significant differences were found in the average number of implantations and numbers of live and resorbed fetuses. Implantation rate was considered as a critical index for the fertility of females [28]. Therefore, it was reasonable for us to conclude that ZYP had no reproductive toxicity in females, owning to no negative effects on the pregnancy and implantation.

There are still some limitations in our study. Considering the combination of ZYP and other medicines, the study on reproductive toxicities of ZYP combined with other medicines is also required. Besides, though ZYP was proofed to be safe for the fertility and early embryonic development in rats, its adverse pregnancy outcomes also should be followed in clinic for thinking of the different sensitivity to Chinese medicine in different species.

According to our results and the analyses above, ZYP had no reproductive toxicity on fertility and early embryonic development in rats. It was concluded that the No Observed Adverse Effect Level (NOAEL) for reproductive toxicity of ZYP on fertility and early embryonic development was equal to or greater than 12 g/kg for rats, which is 48x equivalent clinical doses. However, as 12 g/kg/time is the maximum gavage dosage of ZYP for rats, the real NOAEL is unable to be investigated. Nonetheless, this preclinical safety evaluation enriched our understanding of the risk of ZYP and provides essential basic data for the further analysis for its mechanism and other indications.

## Figures and Tables

**Table 1:** Effects of Zishen Yutai Pill on the fertility.

Terms	Vehicle control group (*n* = 25)	Positive control group (*n* = 25)	Zishen Yutai Pill-treated groups		
			Low dose-treated group (3 g/kg/d, *n* = 25)	Moderate dose-treated group (6 g/kg/d, *n* = 25)	High dose-treated group (12 g/kg/d, *n* = 25)
Females cohabited (*n*)	25	25	25	25	25
Females copulated (*n*)	22	24	25	23	23
Females pregnant (*n*)	20	23	25	21	22
Copulation index (%)	88	96	100	92	92
Fecundity index (%)	90.9	95.8	100	91.3	95.7
Fertility index (%)	80	92	100	84	88
Days of mating period (d)	2.82 ± 1.01	2.92 ± 0.88	2.52 ± 0.92	2.70 ± 1.11	2.74 ± 2.22

Copulation index (%): (number of females copulated/number of females cohabited) × 100.

Fecundity index (%): (number of females pregnant/number of females copulated) × 100.

Fertility index (%): (number of females pregnant/number of females cohabited) × 100.

**Table 2:** Changes in weight of male organs of generation.

Terms	Vehicle control group	Positive control group	Zishen Yutai Pill-treated groups		
			Low dose-treated group	Moderate dose-treated group	High dose-treated group
Body weight (g)	451.9 ± 35.3	331.9 ± 32.4^*∗∗*^	458.3 ± 33.3	448.0 ± 33.4	443.9 ± 35.5
Testis weight (g)	2.993 ± 0.278	2.874 ± 0.189	3.001 ± 0.146	3.033 ± 0.193	3.030 ± 0.165
Testicular coefficient (%)	0.666 ± 0.075	0.874 ± 0.107^*∗∗*^	0.658 ± 0.050	0.680 ± 0.057	0.688 ± 0.074
Epididymis weight (g)	0.912 ± 0.108	0.855 ± 0.101	0.971 ± 0.112	0.971 ± 0.079	0.989 ± 0.147
Epididymis coefficient (%)	0.203 ± 0.026	0.259 ± 0.031^*∗∗*^	0.213 ± 0.029	0.217 ± 0.019	0.224 ± 0.039
Foreskin gland weight (g)	0.069 ± 0.032	0.053 ± 0.018	0.058 ± 0.022	0.068 ± 0.024	0.063 ± 0.019
Foreskin gland coefficient (%)	0.015 ± 0.007	0.016 ± 0.005	0.013 ± 0.005	0.015 ± 0.006	0.014 ± 0.004
Prostate weight (g)	1.030 ± 0.240	0.823 ± 0.174^*∗∗*^	1.064 ± 0.196	0.982 ± 0.226	1.044 ± 0.182
Prostate coefficient (%)	0.230 ± 0.057	0.248 ± 0.050	0.232 ± 0.040	0.220 ± 0.051	0.236 ± 0.044
Seminal vesicle weight (g)	0.984 ± 0.217	0.710 ± 0.197^*∗∗*^	0.890 ± 0.133	0.927 ± 0.230	0.973 ± 0.197
Seminal vesicle coefficient (%)	0.220 ± 0.055	0.213 ± 0.053	0.196 ± 0.036	0.208 ± 0.054	0.220 ± 0.044
Levator ani muscle weight (g)	0.319 ± 0.079	0.238 ± 0.074^*∗∗*^	0.300 ± 0.073	0.281 ± 0.068	0.305 ± 0.073
Levator ani muscle coefficient (%)	0.071 ± 0.018	0.071 ± 0.020	0.065 ± 0.014	0.063 ± 0.014	0.069 ± 0.016
Sphincter weight (g)	1.048 ± 0.143	0.901 ± 0.142^*∗∗*^	1.080 ± 0.189	1.059 ± 0.163	1.064 ± 0.151
Sphincter coefficient (%)	0.233 ± 0.033	0.272 ± 0.035^*∗∗*^	0.236 ± 0.037	0.237 ± 0.039	0.241 ± 0.037

^*∗∗*^ *P* < 0.01 compared with the vehicle control group.

Coefficients of organs: (organ weight/body weight) × 100.

**Table 3:** Changes in epididymal sperm number and motility in male rats.

Terms	Vehicle control group	Positive control group	Zishen Yutai Pill-treated groups		
			Low dose-treated group	Moderate dose-treated group	High dose-treated group
Epididymal weight (g)	0.175 ± 0.033	0.158 ± 0.028	0.180 ± 0.026	0.167 ± 0.028	0.183 ± 0.036
Sperm density (10^6^/mL)	22.3 ± 14.7	12.5 ± 12.6^*∗*^	16.7 ± 9.2	21.4 ± 11.2	18.2 ± 10.7
Motile sperm density (10^6^/mL)	12.9 ± 9.8	6.6 ± 9.9	12.1 ± 8.8	14.5 ± 10.9	11.3 ± 8.8
Progressive sperm density (10^6^/mL)	3.5 ± 2.8	1.9 ± 3.2	3.3 ± 2.2	4.0 ± 2.9	3.3 ± 2.6
Percentage of sperm motility (%)	55.6 ± 24.8	44.4 ± 22.6	66.6 ± 15.7	59.9 ± 26.5	54.5 ± 26.9
Percentage of rapid activities (%)	46.7 ± 23.1	37.7 ± 20.2	58.6 ± 14.4	51.7 ± 23.5	47.5 ± 25.4
Sperm count (10^6^/g)	131.9 ± 94.0	76.9 ± 58.9^*∗*^	94.6 ± 55.4	125.6 ± 53.8	102.2 ± 59.4
Average path velocity (m)	196.5 ± 35.5	201.2 ± 39.3	217.8 ± 20.1	213.8 ± 44.3	211.1 ± 44.1
Straight-line velocity (mm/s)	132.8 ± 23.2	134.3 ± 33.5	147.7 ± 16.6	144.7 ± 33.7	145.2 ± 30.8
Curvilinear velocity (mm/s)	337.1 ± 71.5	349.4 ± 81.3	371.0 ± 45.2	360.9 ± 87.7	357.6 ± 78.7
Amplitude of lateral head displacement (mm)	13.6 ± 3.6	13.2 ± 3.3	14.7 ± 1.9	14.1 ± 4.2	13.9 ± 3.7
Beat-cross frequency (HZ)	16.6 ± 4.4	16.2 ± 4.6	15.8 ± 2.7	17.7 ± 8.0	15.4 ± 4.0
Straightness (%)	67.0 ± 7.7	63.9 ± 5.9	65.3 ± 3.3	64.8 ± 6.4	66.8 ± 3.0
Linearity (%)	43.0 ± 9.4	40.2 ± 6.9	41.4 ± 3.9	41.6 ± 5.2	42.3 ± 3.8
Elongation (%)	29.7 ± 8.1	28.4 ± 13.7	28.0 ± 3.8	31.5 ± 13.9	27.4 ± 5.3
Area (*μ*m^2^)	230.8 ± 49.6	857.8 ± 3251.5	229.9 ± 39.5	881.1 ± 3251.4	221.3 ± 49.3
Abnormal sperm count (*n*/1000)	14.7 ± 1.8	69.7 ± 5.5^*∗∗*^	15.8 ± 1.7	16.3 ± 2.8	16.6 ± 3.6

^*∗*^ *P* < 0.05 and ^*∗∗*^ *P* < 0.01 compared with the vehicle control group.

**Table 4:** Changes in weight and food intake of female rats during the premating and gestation period.

Terms	Days	Vehicle control group	Positive control group	Zishen Yutai Pill-treated groups		
				Low dose-treated group	Moderate dose-treated group	High dose-treated group
Weight (g)						
Premating period	D_1_	210.9 ± 10.5	213.7 ± 11.2	214.1 ± 11.5	212.5 ± 11.1	214.3 ± 11.6
	D_4_	221.8 ± 12.7	212.8 ± 9.6	216.6 ± 14.4	217.3 ± 12.2	218.2 ± 12.4
	D_8_	230.8 ± 17.4	217.7 ± 12.8	223.6 ± 16.2	224.0 ± 13.7	225.1 ± 16.9
	D_11_	231.3 ± 15.7	222.1 ± 11.8	226.6 ± 13.2	225.3 ± 11.6	227.3 ± 13.8
Gestation period	GD_0_	231.4 ± 14.3	225.7 ± 11.2	227.8 ± 13.7	227.2 ± 15.1	232.2 ± 16.5
	GD_3_	247.7 ± 16.7	243.9 ± 10.9	244.1 ± 15.4	244.3 ± 14.8	247.4 ± 13.8
	GD_7_	262.2 ± 16.1	259.5 ± 11.2	258.8 ± 14.6	256.8 ± 16.2	259.0 ± 14.4
	GD_10_	274.4 ± 15.9	270.5 ± 12.2	269.8 ± 14.1	267.7 ± 17.5	272.4 ± 16.2
	GD_14_	291.5 ± 18.8	280.2 ± 21.1	289.5 ± 14.5	287.3 ± 17.7	292.2 ± 17.6
Extra-uterine weight gain		48.7 ± 11.3	43.5 ± 16.1	50.5 ± 5.7	49.5 ± 7.1	48.9 ± 9.0
Food intake (g/d)						
Premating period	D_1_	15.4 ± 1.4	8.2 ± 1.8^*∗∗*^	15.6 ± 0.5	15.1 ± 2.4	14.5 ± 1.5
	D_8_	17.0 ± 1.8	18.7 ± 2.4	18.0 ± 1.9	18.3 ± 0.6	20.4 ± 1.7^*∗*^
Gestation period	GD_0_	17.0 ± 4.2	18.0 ± 4.2	16.0 ± 2.7	16.1 ± 3.2	16.5 ± 2.5
	GD_6_	21.8 ± 2.6	23.6 ± 1.9	22.3 ± 2.0	21.1 ± 3.3	21.0 ± 2.8
	GD_13_	22.3 ± 2.2	24.1 ± 4.0	23.2 ± 2.3	23.1 ± 2.8	23.0 ± 2.5

^*∗*^ *P* < 0.05 and ^*∗∗*^ *P* < 0.01 compared with the vehicle control group.

Extra-uterine weight gain: (weight on GD_14_) − (weight on GD_0_) − (total weight of the gravid uterus).

**Table 5:** Changes in fertility and pregnancy outcome in female rats.

Terms	Vehicle control group	Positive control group	Zishen Yutai Pill-treated groups		
			Low dose-treated group	Moderate dose-treated group	High dose-treated group
Abnormal viscera (*n*)	0	0	0	0	0
Total weight of the gravid uterus (g)	11.4 ± 1.6	11.0 ± 2.2	11.2 ± 1.2	10.5 ± 1.6	11.1 ± 2.6
Ovary weight (g)	0.16 ± 0.02	0.16 ± 0.02	0.16 ± 0.03	0.15 ± 0.02	0.15 ± 0.02
Uterine weight (g)	2.52 ± 0.31	2.38 ± 0.40	2.60 ± 0.26	2.41 ± 0.41	2.53 ± 0.54
Estrous cycle (d)					
5 d before treatment					
Diestrus	3.28 ± 0.61	3.32 ± 0.80	2.96 ± 0.84	3.20 ± 0.82	3.16 ± 0.90
Proestrus	0.44 ± 0.51	0.52 ± 0.65	0.48 ± 0.65	0.48 ± 0.51	0.40 ± 0.50
Estrus	0.88 ± 0.78	0.64 ± 0.64	0.80 ± 0.76	0.72 ± 0.68	0.80 ± 0.76
Postestrus	0.44 ± 0.51	0.52 ± 0.51	0.72 ± 0.46	0.60 ± 0.58	0.64 ± 0.57
14 d after treatment					
Diestrus	13.04 ± 2.96	11.96 ± 3.23	12.44 ± 1.50	12.28 ± 3.25	12.40 ± 3.03
Proestrus	1.60 ± 1.38	2.04 ± 0.98	1.76 ± 0.93	2.12 ± 1.27	2.12 ± 1.30
Estrus	0.96 ± 0.79	1.56 ± 1.19	0.88 ± 0.78	1.16 ± 1.07	1.24 ± 0.93
Postestrus	1.44 ± 1.39	1.80 ± 1.22	1.44 ± 1.19	2.04 ± 1.17	1.88 ± 1.48
Luteal					
Total number of lutea (*n*)	281	303	351	273	298
Average number of lutea (*n*)	14.1 ± 1.3	13.2 ± 1.6	14.0 ± 1.2	13.0 ± 1.8	13.5 ± 1.8
Preimplantation loss rate (%)	4.6	7.6	6.6	6.2	6.4
Implantation					
Implantation number (*n*)	268	280	328	257	279
Average number of implantations (*n*)	13.4 ± 1.1	12.2 ± 2.4	13.1 ± 1.1	12.2 ± 1.9	12.7 ± 2.6
Implantation rate (%)	95.4	92.4	93.4	94.1	93.6
Live fetuses					
Number of litters	20	23	25	21	22
Number of live fetuses (*n*)	250	255	310	242	266
Average number of live fetuses (*n*)	12.5 ± 2.1	11.1 ± 3.5	12.4 ± 1.5	11.5 ± 2.0	12.1 ± 2.7
Live fetuses rate (%)	93.3	91.1	94.5	94.2	95.3
Resorbed fetuses					
Number of litters (*n*)	11	11	10	12	9
Number of resorbed fetuses (*n*)	18	25	18	15	13
Resorbed fetuses rate (%)	6.7	8.9	5.5	5.8	4.7

## References

[1] Gao Q., Han L., Li X., Cai X. (2015). Traditional Chinese medicine, the Zishen Yutai pill, ameliorates precocious endometrial maturation induced by controlled ovarian hyperstimulation and improves uterine receptivity via upregulation of HOXA10. *Evidence-Based Complementary and Alternative Medicine*.

[2] Zhang Y., Liu F. (1983). Clinical review of Zishen Yutai Pill: a report of 150 cases. *New Traditional Medicine*.

[3] Mei X. (2011). The clinical observation of benzene three phenols with zishen yutai pill in treatment of threatened abortion. *Guide of China Medicine*.

[4] Zhao Y., LeiCao, Luo S. (2011). Clinical application and research of Zishen Yutai Pill. *World Chinese Medicine*.

[5] Zheng Z., Jie-Ying L. (2008). Clinical observation on the treatment of luteal phase defect menstrual disorder by zishen yutai pill. *Liaoning Journal of Traditional Chinese Medicine*.

[6] Cakiroglu B., Eyyupoglu S. E., Gozukucuk R., Uyanik B. S. (2014). Ubiquinol effect on sperm parameters in subfertile men who have astheno-teratozoospermia with normal sperm concentration. *Nephro-Urology Monthly*.

[7] Zou Q., Wang R. (2002). Zishen yutai pill. *Guangdong Yao Xue*.

[8] Tao L., Yifeng P., Benhai Y. (2013). Clinical study on the therapy of oligospermia and asthenospermia by zinc gluconate oral liquid and clomiphene. *Anhui Medical Journal*.

[9] Sun Z.-G., Lian F., Zhang J.-W. (2012). Effects of Chinese medicines for tonifying the kidney on multi-fetal pregnancy selective reduction in early stage. *China Journal of Traditional Chinese Medicine and Pharmacy*.

[10] Raine J. M., Nooney J. M. (2015). *A Medicines Regulatory Perspective on Women's Medicines*.

[11] Zhou J., Zhou L., Chong L. (2015). The Zishen Yutai pill shows no reproductive toxicity on embryo-fetal development in rats and rabbits. *Toxicology Research*.

[12] *Drug Reproductive Toxicity Study Technical Guidelines*.

[13] Xu S. (1996). *The Pharmacological Experimental Methodology*.

[14] Fang R., Zhao C. (2008). Clinical verification of Zishen Yutai Pill. *Liaoning Journal of Traditional Chinese Medicine*.

[15] Chabra A., Shokrzadeh M., Naghshvar F., Salehi F., Ahmadi A. (2014). Melatonin ameliorates oxidative stress and reproductive toxicity induced by cyclophosphamide in male mice. *Human & Experimental Toxicology*.

[16] Pendse S., Ginsburg E., Singh A. K. (2004). Strategies for preservation of ovarian and testicular function after immunosuppression. *American Journal of Kidney Diseases*.

[17] Kim W., Kim S.-H., Park S. K., Chang M. S. (2012). Astragalus membranaceus ameliorates reproductive toxicity induced by cyclophosphamide in male mice. *Phytotherapy Research*.

[18] Riaz F., Khan U. A., Ayub M., Shaukat S. (2011). Protective role of ginger on lead induced derangement in plasma testosterone and luteinizing hormone levels of male Sprague Dawley rats. *Journal of Ayub Medical College, Abbottabad*.

[19] Amann R. P. (1981). A critical review of methods for evaluation of spermatogenesis from seminal characteristics. *Journal of Andrology*.

[20] Qiu Y., Wang L.-G., Zhang L.-H., Zhang A.-D., Wang Z.-Y. (2012). Quality of sperm obtained by penile vibratory stimulation and percutaneous vasal sperm aspiration in men with spinal cord injury. *Journal of Andrology*.

[21] Love C. C. (2011). Relationship between sperm motility, morphology and the fertility of stallions. *Theriogenology*.

[22] Jana K., Jana S., Samanta P. K. (2006). Effects of chronic exposure to sodium arsenite on hypothalamo-pituitary-testicular activities in adult rats: possible an estrogenic mode of action. *Reproductive Biology and Endocrinology*.

[23] Leung K. W., Wong A. S. (2014). Ginseng and male reproductive function. *Spermatogenesis*.

[24] Xiao T. T., Xu M., Yang X. H. (2014). The evaluation on embryotoxicity of Dipsaci Radix with mice and embryonic stem cells. *Journal of Ethnopharmacology*.

[25] Zhou J., Qu F. (2009). Treating gynaecological disorders with traditional Chinese medicine: a review. *African Journal of Traditional, Complementary & Alternative Medicines*.

[26] Huo G. (1996). Analysis and evaluation of amino acids and mineral composition in Colla coriiasini. *Amino Acids & Biotic Resources*.

[27] Liu H., Qiu N., Ding H., Yao R. (2008). Polyphenols contents and antioxidant capacity of 68 Chinese herbals suitable for medical or food uses. *Food Research International*.

[28] Perobelli J. E., Alves T. R., de Toledo F. C. (2012). Impairment on sperm quality and fertility of adult rats after antiandrogen exposure during prepuberty. *Reproductive Toxicology*.

[29] Yu Y., Yang A., Zhang J., Hu S. (2013). Maternal exposure to the mixture of organophosphorus pesticides induces reproductive dysfunction in the offspring. *Environmental Toxicology*.

[30] Bittner A.-K., Horsthemke B., Winterhager E., Grümmer R. (2011). Hormone-induced delayed ovulation affects early embryonic development. *Fertility and Sterility*.

[31] Bhat G., Dhaliwal G., Ghuman S., Honparkhe M. (2014). Effect of human chorionic gonadotropin on post-ovulatory mid-luteal profile and subsequent pregnancy rate in anestrus buffalo using two synchronization regimes. *Applied Biological Research*.

[32] Zhou Y., Zhang H., He J. (2013). Effects of sodium fluoride on reproductive function in female rats. *Food and Chemical Toxicology*.

